# Extract of a Letter from William B. Diver to Professor Dunglison

**Published:** 1845-12

**Authors:** 


*Extract of a Letter from* Dr. William B. Diver *to* Professor Dunglison, *dated Cincinnati,* Oct. 25th, 1845.

Dear Sir,—This morning my attention was directed by one of my pupils in the dissecting room, to a peculiar abnormal position of one of the kidneys in a subject brought for dissection. The kidney, which should have been found in the left lumbar region, was situate in the pelvis, immediately below the promontory of the sacrum. It was much smaller than the kidney of the right side, and derived its artery from the common iliac of the *right side,* about half an inch below the bifurcation. A deep sulcus was formed upon its surface for the passage of the renal artery. The convexity looked upwards, and the pelvis was much distended, owing perhaps to the gravitation of the urine. The distribution of the renal artery is very remarkable in this case, arising as it does from the common iliac of the *right side.* Another peculiarity in the distribution of the branches of the abdominal aorta was, an arterial branch corresponding nearly in its origin to the inferior mesenteric, but which took a course along the lumbar vertebra without giving off any branches until it dipped down behind the bladder. This malposition of the kidney was witnessed by several physicians of the city, and, I presume, is not devoid of interest to you as a physiologist and a public teacher. I was consulted at the Northern Dispensary by an old man respecting his oldest daughter, an unmarried person, who had attained the age of 43 without menstruating. At that time the menses appeared with great disturbance of the general health.

Since I last wrote you I have used the emplast. iod. in a case of lymphatic tumour of the neck unconnected with scrofulous diathesis, with perfect success; and in a case of indurated mammæ following abortion in the fifth month, with entire satisfaction.

